# Older Age, a High Titre of Neutralising Antibodies and Therapy with Conventional DMARDs Are Associated with Protection from Breakthrough Infection in Rheumatoid Arthritis Patients after the Booster Dose of Anti-SARS-CoV-2 Vaccine

**DOI:** 10.3390/vaccines11111684

**Published:** 2023-11-02

**Authors:** Andrea Picchianti-Diamanti, Assunta Navarra, Alessandra Aiello, Bruno Laganà, Gilda Cuzzi, Andrea Salmi, Valentina Vanini, Fabrizio Maggi, Silvia Meschi, Giulia Matusali, Stefania Notari, Chiara Agrati, Simonetta Salemi, Roberta Di Rosa, Damiano Passarini, Valeria Di Gioia, Giorgio Sesti, Fabrizio Conti, Francesca Romana Spinelli, Angela Corpolongo, Maria Sole Chimenti, Mario Ferraioli, Gian Domenico Sebastiani, Maurizio Benucci, Francesca Li Gobbi, Anna Paola Santoro, Andrea Capri, Vincenzo Puro, Emanuele Nicastri, Delia Goletti

**Affiliations:** 1 Department of Clinical and Molecular Medicine, “Sapienza” University, S. Andrea University Hospital, 00189 Rome, Italy; andrea.picchiantidiamanti@uniroma1.it (A.P.-D.); bruno.lagana@uniroma1.it (B.L.); simonettasalemi@gmail.com (S.S.); roberta.dirosa@uniroma1.it (R.D.R.); passarini.1694998@studenti.uniroma1.it (D.P.); valeria.digioia@hotmail.it (V.D.G.); giorgio.sesti@uniroma1.it (G.S.); 2 Epidemiology Department, National Institute for Infectious Diseases Lazzaro Spallanzani—Istituto di Ricovero e Cura a Carattere Scientifico (IRCCS), 00149 Rome, Italy; assunta.navarra@inmi.it; 3 Translational Research Unit, National Institute for Infectious Diseases Lazzaro Spallanzani—Istituto di Ricovero e Cura a Carattere Scientifico (IRCCS), 00149 Rome, Italy; alessandra.aiello@inmi.it (A.A.); gilda.cuzzi@inmi.it (G.C.); andrea.salmi@inmi.it (A.S.); valentina.vanini@inmi.it (V.V.); 4 Unità Operativa Semplice (UOS) Professioni Sanitarie Tecniche, National Institute for Infectious Diseases Lazzaro Spallanzani—Istituto di Ricovero e Cura a Carattere Scientifico (IRCCS), 00149 Rome, Italy; 5 Laboratory of Virology, National Institute for Infectious Diseases Lazzaro Spallanzani—Istituto di Ricovero e Cura a Carattere Scientifico (IRCCS), 00149 Rome, Italy; fabrizio.maggi@inmi.it (F.M.); silvia.meschi@inmi.it (S.M.); giulia.matusali@inmi.it (G.M.); 6 Laboratory of Cellular Immunology and Clinical Pharmacology, National Institute for Infectious Diseases Lazzaro Spallanzani—Istituto di Ricovero e Cura a Carattere Scientifico (IRCCS), 00149 Rome, Italy; stefania.notari@inmi.it (S.N.); chiara.agrati@opbg.net (C.A.); 7 Department of Pediatric Hematology and Oncology, Bambino Gesù Pediatric Hospital, 00152 Rome, Italy; 8 Reumatologia, Dipartimento di Scienze Cliniche Internistiche, Anestesiologiche e Cardiovascolari, “Sapienza” Università di Roma, 00161 Rome, Italy; fabrizio.conti@uniroma1.it (F.C.); francescaromana.spinelli@uniroma1.it (F.R.S.); 9 Clinical Division of Infectious Diseases, National Institute for Infectious Diseases Lazzaro Spallanzani—Istituto di Ricovero e Cura a Carattere Scientifico (IRCCS), 00149 Rome, Italy; angela.corpolongo@inmi.it (A.C.); emanuele.nicastri@inmi.it (E.N.); 10 Rheumatology, Allergology and Clinical Immunology, Department of ‘Medicina dei Sistemi’, University of Rome ‘Tor Vergata’, 00133 Rome, Italy; maria.sole.chimenti@uniroma2.it; 11 Department of Rheumatology, San Camillo Hospital, 00152 Rome, Italy; ferraioli.mar@gmail.com (M.F.); gsebastiani@scamilloforlanini.rm.it (G.D.S.); 12 Rheumatology Unit, S. Giovanni di Dio Hospital, Azienda USL—Toscana Centro, 50122 Florence, Italy; maurizio.benucci@uslcentro.toscana.it (M.B.); francesca.ligobbi@uslcentro.toscana.it (F.L.G.); 13 UOC Emerging Infections and Centro di Riferimento AIDS (CRAIDS), National Institute for Infectious Diseases Lazzaro Spallanzani—Istituto di Ricovero e Cura a Carattere Scientifico (IRCCS), 00149 Rome, Italy; annapaola.santoro@inmi.it (A.P.S.); andrea.capri@inmi.it (A.C.); vincenzo.puro@inmi.it (V.P.); 14 Istituto di Ricovero e Cura a Carattere Scientifico (IRCCS), Bambino Gesù Children’s Hospital, 00165 Rome, Italy

**Keywords:** SARS-CoV-2, vaccine, rheumatoid arthritis, immunogenicity, neutralising antibodies, immunosuppressive therapy, booster, protective immunity

## Abstract

*Objectives*: We aimed to analyse the incidence and severity of breakthrough infections (BIs) in rheumatoid arthritis (RA) patients after a COronaVIrus Disease 2019 (COVID-19) vaccination booster dose. *Methods*: We enrolled 194 RA patients and 1002 healthcare workers (HCWs) as controls. Clinical, lifestyle and demographic factors were collected at the time of the third dose, and immunogenicity analyses were carried out in a subgroup of patients at 4–6 weeks after the third dose. *Results:* BIs were experienced by 42% patients (82/194) with a median time since the last vaccination of 176 days. Older age (>50 years; aHR 0.38, 95% CI: 0.20–0.74), receiving conventional synthetic disease modifying antirheumatic drugs (csDMARDs) (aHR 0.52, 95%CI: 0.30–0.90) and having a titre of neutralising antibodies >20 (aHR 0.36, 95% CI: 0.12–1.07) were identified as protective factors. Conversely, anti-IL6R treatment and anti-CD20 therapy increased BI probability. BIs were mostly pauci-symptomatic, but the hospitalisation incidence was significantly higher than in HCWs (8.5% vs. 0.19%); the main risk factor was anti-CD20 therapy. *Conclusions:* Being older than 50 years and receiving csDMARDs were shown to be protective factors for BI, whereas anti-IL6R or anti-CD20 therapy increased the risk. Higher neutralising antibody titres were associated with a lower probability of BI. If confirmed in a larger population, the identification of a protective cut-off would allow a personalised risk–benefit therapeutic management of RA patients.

## 1. Introduction

The worldwide vaccination campaign against COronaVIrus Disease-2019 (COVID-19) was demonstrated to be highly effective in preventing Severe Acute Respiratory Syndrome Coronavirus 2 (SARS-CoV-2) infection and reducing the risk of COVID-19 severity and complications, allowing the end of the pandemic state to be declared 30 months after its introduction [1].

The initial phase of the vaccine campaign started worldwide at the beginning of 2021, with the first generation of mRNA and adenoviral vaccines directed against the ancestral SARS-CoV-2 strain. From November 2021, the scenario of the SARS-CoV-2 pandemic changed with the emergence of the Omicron variant of concern (VOC), characterised by a higher number of mutations in the spike protein compared to any other variant [2]. Omicron became predominant worldwide, and in Italy, in less than 1 month after its first detection, on 3 January 2022 represented the most predominant VOC (76.9–80.2%) of SARS-CoV-2 infections [3,4]. Aware of the waning of immunity and the large spread of Omicron, the regulatory agencies suggested a third dose of vaccination, starting with the most immunologically fragile subjects [5,6,7].

The management of patients affected by Autoimmune Rheumatic Diseases (ARDs) during the COVID-19 pandemic was challenging for both patients and healthcare providers due to the complex and interwoven link between the infection autoimmunity and immunosuppressive treatments. Patients with ARDs under immunosuppressive therapy had priority access to COVID-19 vaccines since they are considered a vulnerable population. Indeed, a moderately increased incidence and severity of SARS-CoV-2 infection have been found in this population, mainly driven by general risk factors such as older age and comorbidities and/or by ongoing immunosuppressive therapies such as anti-CD20 monoclonal antibody treatment [8,9,10].

Evidence indicated that the first vaccine cycle induced anti-receptor-binding domain (RBD) and neutralising antibodies and a specific T-cell response in the majority of ARD patients, although with a different grade depending on the immune suppressive therapy used [11,12,13].

Although the Omicron variant can escape the protection generated by the two doses of COVID-19 vaccines based on vector or mRNA technology, a three-dose mRNA vaccination was shown to increase immunity against this variant and to reduce the incidence and severity of breakthrough infections (BI) in the general population [14,15,16,17,18]; however, this evidence in ARD patients is scarce. Furthermore, most of the studies are focused on immunogenicity data as a surrogate for protection, but no clear association between humoral or T cell immunity induced by vaccination and the risk of BI and complications has been described so far [19,20,21]. These immune markers would be relevant in finding correlates of protection, especially for vulnerable populations such as ARDs under immunosuppressive treatments.

Therefore, this study aimed to evaluate the incidence and severity of SARS-CoV-2 BI in COVID-19-free rheumatoid arthritis (RA) patients after the booster dose vaccination in comparison with a large control group of COVID-19-free healthcare workers (HCWs). We also evaluated clinical and demographic characteristics associated with BI and specific B and T cell immunity induced by vaccines.

## 2. Materials and Methods

### 2.1. Study Population and Design

This is a prospective, real-life, multicentre study. Patients with an RA diagnosis according to the 2010 criteria of the European League Against Rheumatism/American College of Rheumatology (EULAR/ACR) [22], under immunosuppressive treatment, were consecutively enrolled at S. Andrea University Hospital (Rome), AO San Camillo Forlanini (Rome), Policlinico Umberto I University Hospital (Rome), Policlinico Tor Vergata University Hospital (Rome) and S. Giovanni di Dio Hospital (Florence). Healthcare workers (HCWs) were recruited at the National Institute for Infectious Diseases (INMI) Lazzaro Spallanzani-Istituto di Ricovero e Cura a Carattere Scientifico (IRCCS). Some subjects were included in our previous studies [23,24]. The protocol was approved by the Lazzaro Spallanzani-IRCCS Ethical Committee (Approval number 297/2021, 318/2021 and 452/2021), and written informed consent was signed by all the patients and HCWs.

Participants who had completed a three-dose schedule of BNT162b2 mRNA vaccine during March 2022 and without previous SARS-CoV-2 diagnosis (i.e., positive to the antigenic and/or molecular test by real-time polymerase chain reaction (PCR) on the swab sample and/or positive to anti-N) were included. We defined a third dose vaccination breakthrough case as a rapid antigen and/or real-time PCR positive nasal or nasopharyngeal sample obtained at least 7 days after the booster vaccine dose. BI was also classified according to its severity: asymptomatic, pauci-symptomatic (i.e., fever, cough, arthromyalgia) or severe (requiring hospitalisation). Several clinical, lifestyle and demographic factors were collected at the time of the third dose; blood samples for immunogenicity analysis were collected 4–6 weeks after the third dose. To evaluate BI risk, patients were followed up until a positive SARS-CoV-2 test, or until 15 February 2023.

### 2.2. Study Procedures

From RA cohorts previously established, a subgroup of 41 RA patients underwent sample collection for the evaluation of the immune-specific response after 4–6 weeks from the booster dose (T2) (Supplementary Figure S1).

We followed standardised protocols for blood drawing and laboratory procedures [25,26]. Blood was collected in lithium heparinised tubes (BD Vacutainer, Becton Dickinson, Florence, Italy, Cat. 367526) and processed within 4 h of collection. Lymphocyte counts were obtained within 1 week from the time of blood sampling.

### 2.3. Anti-SARS-CoV-2 Antibody Testing

Both anti-nucleoprotein immunoglobulin G (Anti-N-IgG) and anti-receptor-binding domain (RBD)-IgG were evaluated following the manufacturer’s instructions (Architect^®^ i2000sr Abbott Diagnostics, Chicago, IL, USA). Anti-N-IgG was expressed as index values [(sample (S)/Cutoff (CO)] and scored positive if titres ≥1.4. We expressed anti-RBD-IgG as binding antibody units (BAU)/mL, and they were considered positive if ≥7.1.

To evaluate the neutralising antibodies, we performed a micro-neutralisation assay (MNA), as previously described [26], using the SARS-CoV-2/Human/ITA/PAVIA10734/2020 (isolated in March and provided by Fausto Baldanti, Pavia, Italy). We reported the neutralisation titre as the reciprocal of the highest serum dilution (MNA_90_) inhibiting at least 90% of the cytopathic effect (CPE). We tested the first dilution and set the threshold at 1:10.

### 2.4. IFN-γ-Specific T-Cell Response against SARS-CoV-2

We evaluated the IFN-γ-specific T-cell response against SARS-CoV-2 using a whole blood platform. The blood was stimulated with a peptide mix (spike) consisting of equal amounts of the three peptide pools at a final concentration of 0.1 µg/mL that covered the whole sequence of SARS-CoV-2 Wuhan spike protein (PepTivator^®^Prot_S1, Prot_S, and Prot_S+, Miltenyi Biotec, Bergisch Gladbach, Germany, Cat. 130–127–048, Cat. 130–126–701 and Cat. 130–127–312, respectively), and was incubated for 16–24 h at 37 °C, as previously shown [25]. As a positive control, blood was stimulated with 200 ng/mL of staphylococcal enterotoxin B (SEB) (Merck Life Science, Milan, Italy, Cat. S4881). After incubation, plasma was collected and stored at −20 °C or −80 °C for subsequent analysis. The ELLA Simple Plex Human IFN-γ (3rd Gen.) Assay (Bio-Techne, Minneapolis, MN, USA, Cat. SPCKB-PS-002574) was used to quantify the IFN-γ levels from which the unstimulated control value was subtracted. The detection limit of the assay was 0.17 pg/mL. The IFN-γ response was scored positive if ≥16 pg/mL [13].

### 2.5. Statistical Analysis

Data were analysed using Stata (StataCorp. 2021. Stata Statistical Software: Release 17. College Station, TX, USA: StataCorp LLC.) and R Project Software (version 4.2.1).

Categorical variables were summarised as absolute and relative percentages, while quantitative variables were summarised as medians and interquartile ranges. Comparisons between independent groups were made using a chi-squared test, Fisher’s exact test or Mann–Whitney test, as appropriate.

Receiver operating curve (ROC) and the area under the curve (AUC) were used to establish the optimal cut-off values of immune response measurements able to discriminate patients with breakthrough infection from those without.

Unadjusted Kaplan–Meier curves were used to visualise the probability of being diagnosed with SARS-CoV-2 infection by days from (i) third dose administration and (ii) the immune response measurement, and differences among groups were tested using the log-rank test.

Univariable proportional hazard Cox regressions were performed to estimate the hazard ratios of being diagnosed with SARS-CoV-2 infection, according to factors analysed after the third dose administration and the immune response measurement. Factors with *p* < 0.2 at the univariable were entered into the respective multivariable model. We identified the factors associated with a lower risk of infection using statistical analysis, and we defined these factors as protective. In Cox models, the proportional hazards assumption was tested using the Schoenfeld residuals. In the case of non-proportional hazards, a weighted Cox regression was conducted to evaluate whether estimates substantially confirmed those obtained from standard Cox regression [27]. A *p*-value less than 0.05 was considered statistically significant. 

## 3. Results

### 3.1. Patients

Within a cohort of 250 RA patients, we enrolled 194 patients who satisfied the inclusion criteria. The demographic, clinical and lifestyle characteristics of the patients are described in Table 1. As a control group, we evaluated 1002 HCWs.

All RA patients were under immunosuppressive therapy: 32% were receiving only conventional synthetic disease-modifying antirheumatic drugs (csDMARDs), 20.1% Tumour Necrosis Factor (TNF)-α inhibitors, 19.6% Cytotoxic T-Lymphocyte Antigen 4-Immunoglobulin (CTLA4-Ig), 9.3% anti-IL6R monoclonal antibody, 25.3% Janus kinase (JAK) inhibitors, 5.7% anti-CD20 monoclonal antibody and 17.1% glucocorticoids (GCs).

### 3.2. Risk of Breakthrough Infections and Clinical Parameters

**Figure 1:**
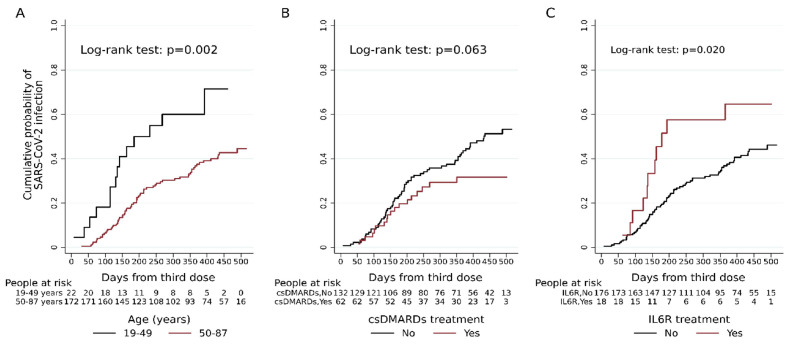
Unadjusted Kaplan-Meier curves estimating the cumulative probability of SARS-CoV-2 infection after third-dose vaccination among 194 RA patients according to the age group (**A**), conventional synthetic disease-modifying antirheumatic drugs (csDMARDs) (**B**) and anti-IL6R treatment (**C**).

Among the RA patients, 42.3% (82/194) had BI after a median time since last vaccination of 176 days [interquartile range (IQR): 118–268]; 26.8% (22/82) patients received early therapy with antivirals or monoclonal antibodies (Table 1). As shown in the Kaplan–Meier curves, subjects younger than 50 years had a significantly higher probability of BI compared to the older subjects (*p =* 0.002) (Figure 1A), whereas subjects taking only csDMARDs had a lower probability of BI (*p =* 0.063) (Figure 1B); on the other hand, anti-IL6R increased the probability of BI (*p =* 0.020) (Figure 1C).

Using univariable proportional hazard Cox regression, we show that smoking habit [hazard ratio (HR) 1.68, 95%confidence interval (CI) 1.02–2.76; *p =* 0.040] and therapy with anti-IL6R (HR 2.09, 95%CI 1.11–3.95; *p =* 0.023) or anti-CD20 monoclonal antibody (HR 1.74, 95%CI 0.80–3.78; *p =* 0.162*)* were directly associated with BI; conversely, having at least one comorbidity (HR 0.59, 95%CI 0.38–0.91; *p =* 0.018) and body mass index (BMI) (HR 0.94, 95%CI 0.89–1.00; *p =* 0.050*)* were inversely associated with BI occurrence.

After including all factors associated with BI in the multivariable model, younger age was confirmed as the main risk factor for BI. Indeed, patients older than 50 years had a risk reduction of 62% [adjusted HR (aHR):0.38, 95% CI: 0.20–0.74, *p =* 0.004] compared to younger subjects. Furthermore, patients treated with csDMARDs showed a 48% reduced risk of BI (aHR: 0.52, 95%CI: 0.30–0.90, *p =* 0.021), whereas the risk of BI doubled in patients treated with anti-IL6R (aHR: 2.01, 95% CI: 1.03–3.89, *p =* 0.039) and nearly tripled in patients treated with anti-CD20 treatment (aHR: 2.88, 95% CI: 1.27–6.51, *p =* 0.011) (Table 2).

### 3.3. Risk of Breakthrough Infection and B and T Cell Immune-Specific Responses

A subgroup of 41 RA patients were evaluated for the immune-specific response after the third vaccine dose. Out of these 41 patients, 39% (n = 16) had a BI during the follow-up period.

RA patients with BI showed lower median titres of anti-S/RBD-IgG (BAU/mL) [476 (IQR: 51–2020) vs. 1360 (IQR: 642–3469) (*p =* 0.145)], and a significantly lower median titre of neutralising antibodies [at 20 (IQR: 5–80) vs. 160 (IQR:40–320) (*p =* 0.005)] than uninfected patients, but similar levels of IFN-γ [at 11 pg/mL (IQR: 1–111) vs. 27 pg/mL (IQR: 4–92) (*p =* 0.446)] (Supplementary Figure S2).

The ROC analysis showed that the cut-offs discriminating BI from non-infection were 809 BAU/mL for anti-S/RBD-IgG, 20 for neutralising antibodies and 16 pg/mL for the IFN-γ response (Supplementary Figure S3).

**Figure 2:**
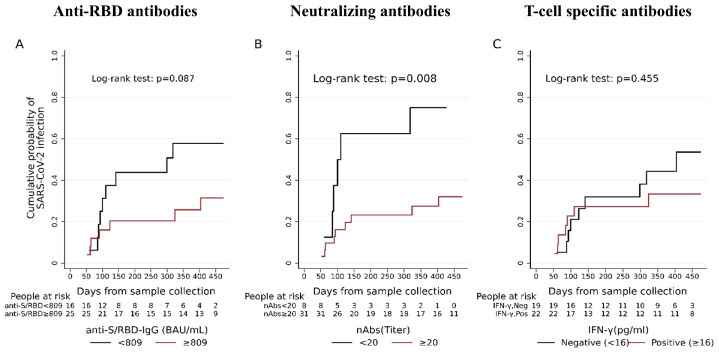
Unadjusted Kaplan-Meier curves estimating the probability of SARS-CoV-2 infection in RA patients according to anti-S/RBD-IgG in binding antibody units (BAU) (**A**), neutralising antibodies (nAbs) (**B**) and T cell-specific response evaluated with IFN-γ detection in picograms (**C**). Abbreviations: anti-S/RBD: anti-spike receptor binding domain; BAU: binding antibody units; nAbs: neutralising antibodies; IFN: interferon; pg: picograms.

In time to event univariable analysis, after sample collection, we found a significantly higher probability of BI for patients with neutralising antibodies lower than 20 (*p =* 0.008), whereas no significant differences were found for anti-S/RBD antibodies lower than 809 BAU/mL (*p =* 0.087) and for IFN-γ levels (*p =* 0.455) (Figure 2 and Table 3).

Accounting for different exposure times to SARS-CoV-2, in the Cox regression models we adjusted the hazard ratios relative to the analysis of immune factors associated with BI for the days elapsed from the third dose to sample collection (median 28 days, IQR: 28–73. Having a higher titre of neutralising antibodies (≥20) still conferred a 64% reduction in the risk of BI (aHR: 0.36, 95% CI: 0.12–1.07, *p =* 0.067), albeit beyond the threshold of significance. Conversely, no significant associations were confirmed between BI and the titre of anti-RBD antibodies or IFN-γ (*p =* 0.346 and 0.906, respectively) (Table 3).

### 3.4. Severity of Breakthrough Infection

Concerning the severity of BI, 10% (8/82) of patients were asymptomatic, 82% (67/82) pauci-symptomatic and 8.5% (7/82) were hospitalised (Table 1).

Anti-CD20 therapy (*p* = 0.001) and a longer disease duration (*p* = 0.045) were the only baseline characteristics significantly associated with BI hospitalisation among the 82 infected RA patients (Table 4).

### 3.5. Comparison between the Risk of Breakthrough Infection in RA and HCWs

Compared to HCWs, RA patients were older (*p* < 0.001), more often had flu vaccination (*p* < 0.001), and received Comirnaty more frequently as the third vaccine dose (*p* < 0.001) (Table 5).

After univariable analysis, RA showed a lower risk of BI (HR 0.74, 95%CI 0.59–0.93, *p =* 0.012); age was inversely associated with BI (HR 0.88, 95%CI 0.83–0.93, *p =* 0.000) and males showed a lower risk of BI compared to females (HR 0.77, 95%CI 0.64–0.91, *p =* 0.003). In the multivariable model, only age and male gender remained inversely associated with BI (*p =* 0.001 and *p =* 0.007, respectively) (Table 6).

The incidence of hospitalisation was 8.5% (7/82) in RA patients compared to that in the HCWs, which was 0.19% (1/530), and this difference was significant (two-sample test of proportions, *p <* 0.001).

## 4. Discussion

A huge number of studies have evaluated the immunogenicity of the SARS-CoV-2 vaccine in ARD patients under immunosuppressive therapy so far [11,12,13]. The results of previous studies are difficult to compare, and partly contradictory because they are affected by heterogenicity in the selected population and immunological analysis; moreover, previous data are mainly restricted to the second vaccine dose. Despite these limits, they have generally shown that the COVID-19 vaccine is immunogenic in most patients with ARD, and different factors associated with a blunted vaccine response have been identified, such as anti-CD20 or CTLA4-Ig therapy [11,12,13].

A higher immune response to vaccination should confer a higher protection against BI and infection complications; however, no clear association between humoral or T cell immunity induced by vaccination and the risk of BI has been identified so far, either in ARD or in the general population. For this reason, current recommendations advise against routine antibody testing after COVID-19 vaccination in immuno-competent individuals [28].

On the other hand, detecting clinical and immune markers of vaccine efficacy in reducing the risk of BI and complications is needed to optimise the management of vulnerable populations such as ARD patients under immunosuppressive therapy. Indeed, identifying patients at risk would enable a better definition of who would benefit from pre-exposure prophylaxis with anti-SARS-CoV-2 monoclonal antibodies, or when withdrawing or changing the ongoing immunosuppressive therapy.

Surprisingly, data focused on the cumulative incidence and severity of BI in ARD patients after the third vaccine dose, and the role played by clinical characteristics, ongoing immune suppressive therapy and immune-specific antibodies and T cell responses on vaccine effectiveness are limited.

In this study, we prospectively evaluated 194 RA patients under immunosuppressive therapy and 1002 HCWs from the time of the booster dose to BI or at least 1 year follow-up. We found that 42% of patients (82/194) presented a BI vs. 53% (530/1002) in the HCWs.

This relatively high rate of BI is likely due to the high diffusion of Omicron VOC. Indeed, the follow-up period of the study overlapped with the spread of this variant, which has a well-known ability to impair the effectiveness of the first-generation COVID-19 vaccines based on the ancestral viral strain.

Interestingly, being older than 50 years and receiving csDMARDs were protective factors for BI, whereas treatment with anti-CD20 and, to a lesser extent, anti-IL-6R therapy significantly increased the probability of BI, as confirmed by the multivariable analysis.

Most BI patients were pauci-symptomatic, but the incidence of hospitalisation was significantly higher than in HCWs (8.5% vs. 0.19%) and the main factor associated with the severity of BI was the use of anti-CD20 therapy.

Data from registries before the Omicron wave showed a very low incidence of BI in patients with ARDs. In particular, the EULAR Coronavirus Vaccine (COVAX) registry reported a BI incidence of 0.7% in ARDs patients with two doses of vaccine [29]. Other studies focused on the first two vaccine doses showed that RA patients still exhibited increased rates of BI and complications compared with those without RA [30]. Li Hui et al., in a large retrospective cohort, reported a higher risk of SARS-CoV-2 infection and COVID-19 hospitalisation in RA patients than the general population after receiving two doses of COVID-19 vaccines over 9 months follow-up (4.17 vs. 3.96/1000) [31]. A nationwide Danish cohort study found that vaccinated RA patients had a lower risk of hospitalisation compared to unvaccinated patients [aHR 1.22 (95% CI 1.09–1.57) vs. 1.09 (0.92–1.14)], but not compared to healthy controls [32]. More recently, an interesting retrospective cohort study evaluated temporal trends in the incidence and severity of COVID-19 among patients with different ARDs from the first wave through the initial Omicron wave and reported data in line with our results [33]. Indeed, these authors found 51% of BI in patients with three vaccine doses during the initial Omicron wave. On the other hand, they reported a higher percentage of COVID-19 hospitalisation than that observed in our study (14.7 vs. 8.5), but it must be considered that it was calculated in a mixed cohort including unvaccinated, partially vaccinated and three-dose-vaccinated patients.

Younger age has been previously demonstrated to be associated with an increased risk of BI in the general population, likely associated with the higher degree of socialisation and behavioural risk factors present among younger people [23,34].

Different studies have already shown that the use of anti-CD20 therapy induces a significant reduction in the humoral response to COVID-19 vaccination and is associated with a higher risk of both SARS-CoV-2 infection and clinical severity [35,36,37,38].

It may be hypothesised that the reduced risk of BI observed in patients receiving csDMARDs is related to the lower immunosuppressive effect and infection risk of these agents compared to biological DMARDs (bDMARDs). Indeed, data on the risk of common infections such as influenza and pneumococcus in ARD patients generally showed that csDMARDs (mainly methotrexate) are relatively safe [39,40,41].

In several studies, it has previously been shown that methotrexate (mtx) does not have any impact on vaccine immunogenicity, in particular after influenza vaccination [42], although more recent data showed that a temporary discontinuation of mtx is significantly associated with increased immunogenicity in patients with RA [43,44].

Regarding the response to COVID-19 vaccines, the literature data are contrasting. Abhishek A et al. reported that withholding mtx enhances the specific antibody responses to COVID-19 vaccination, as shown with influenza vaccines [45,46]. However, a systematic review did not find any significant reduction in the humoral response in patients receiving mtx [47]. The mild reduction in the antibody response to vaccines is likely due to the indirect effects of mtx on B cells. Indeed, it has been reported that mtx in the presence of high B lymphocyte stimulating factor (Blys) levels negatively impacts the vaccine response to seasonal influenza vaccination, probably by inducing adenosine release from B cells and increasing B regulatory cells, as previously observed in BLyS tg mice immunised with TNF-inhibitors [43,48].

It is difficult to provide an interpretation of the unexpectedly higher risk of BI that emerged in patients under anti-IL6R treatment. Indeed, the impact of this agent on immunogenicity to the COVID-19 vaccine is debated [13,49], and to our knowledge there are no other studies focused on its role on vaccine effectiveness. It is likely, since IL-6 involves a physiological modulation of B cells, that this may have had an impact on BI risk. Indeed, IL-6 acts on the follicular helper T cells promoting B cell proliferation and immunoglobulin class switching, thus playing an important role in controlling the survival and maturation of B cells and plasmablasts [50,51]. In addition, anti-IL6R are generally used after the failure of previous bDMARDs (i.e., TNF-α inhibitors), thus in patients with more severe and “difficult-to-treat” disease, and consequently with a higher dysregulation of the immune system and susceptibility to infections. However, the small sample size of patients receiving this agent cannot allow for firm conclusions.

In the subgroup of patients with immunological data, we observed a higher probability of BI in patients with titres of anti-S/RBD antibodies less than 809 BAU/mL and a neutralising titre less than 20. However, when these data were adjusted for days elapsed from the third dose to sample collection, interestingly, only the titre of neutralising antibodies still conferred a 64% reduction in the risk of BI.

As far as we know, only one other work has assessed the potential correlation between the incidence of BI and immunogenicity response in ARD patients after receiving the third dose of anti-SARS-CoV-2 vaccine [52], demonstrating that a significant lower neutralising response was associated with BI after the third vaccine dose.

Compared to HCWs, at univariable analysis RA patients showed a lower risk of BI, age was inversely associated with BI and males showed a lower risk of BI compared to females. However, in the multivariable model, only age and male gender remained inversely associated with BI.

Although HCWs and RA subjects were almost comparable in terms of BI risk, the RA patients showed a higher incidence of hospitalisation. These data support the concept that ARD patients under immunosuppressive treatment are broadly protected by the mRNA vaccine, but still belong to a vulnerable population that needs careful attention. In this context, the identification of risk factors for BI and severity is of the utmost importance to optimise the management of these patients.

Some limitations of this study need to be considered. First, immunogenicity data were available only for a subgroup of patients; however, the results seem robust because they were also confirmed by the multivariate analysis. Moreover, considering the real-life design of the study, we may not rule out potential reporting errors; however, the data collection was internally checked by the investigators and then further by those performing the data analysis.

Conversely, this study has several strengths, being multicentre, prospective, with a long-term follow-up design, with the presence of a homogeneous and deeply characterised cohort of RA patients, with immunogenicity data from B and T cell compartments in a subgroup of RA subjects and having a large control group of HCWs.

## 5. Conclusions

To the best of our knowledge, these are the first real-world data on the incidence and severity of BI and its correlation with clinical and immunogenicity findings after the third dose of COVID-19 vaccine in RA patients. Our results suggest that being older than 50 years and receiving csDMARDs are protective factors for BI, whereas treatment with anti-IL6R and anti-CD20 therapy significantly increased the probability of BI. Patients with a neutralising titre over 20 had a lower probability of BI, with a 64% reduction in the risk when data were adjusted for days elapsed from the third dose to sample collection. If confirmed in a larger population, the identification of this protective cut off would be of great clinical relevance, thus allowing the personalised risk–benefit therapeutic management of ARD patients.

## Figures and Tables

**Table 1:** Demographic and clinical characteristics of the 194 rheumatoid arthritis patients.

Patients’ Characteristics	RA Patients
**Total**	194 (100)
**Age**, median (IQR) years	63 (54–71)
**Age class, *n* (%)**	
19–49	22 (11.3)
50–59	49 (25.3)
60–69	66 (34.0)
70–87	57 (29.4)
**Gender**: Female, ***n* (%)**	137 (70.6)
**Presence of comorbidities, *n* (%)**	130 (67.0)
No	64 (33.0)
Yes, one	78 (40.2)
Yes, two or more	52 (26.8)
**BMI**, median (IQR)	26.9 (23.3–28.4)
**Smoking habits, *n* (%)**	39 (20.1)
**Flu vaccination (2021/2022), *n* (%)**	112 (57.7)
**Vaccination booster type, *n* (%)**	
Comirnaty	190 (97.9)
Spikevax/other	4 (2.1)
**Rheumatoid arthritis disease**	
**Disease duration**, median (IQR) months	120 (72–186)
**Immunosuppressive drugs; regimens containing:**	
Conventional DMARDs, *n* (%)	62 (32.0)
TNFα-i, *n* (%)	39 (20.1)
IL6R-i, *n* (%)	18 (9.3)
CTLA4-Ig, *n* (%)	38 (19.6)
JAK-i, *n* (%)	49 (25.3)
CD20-i, *n* (%)	11 (5.7)
Corticosteroids, *n* (%)	33 (17.1)
**Immunogenicity data**, Samples available, ***n* (%)**	41 (21.1)
Days from booster dose to sample, median (IQR)	28 (28–73)
**Among 82 RA patients with breakthrough infection**	
Days from 3rd dose to infection, median (IQR)	176 (118–268)
**Early therapy with antivirals or monoclonal antibodies, *n* (%)**	
Yes	22 (26.8)
No	60 (73.2)
**Severity of COVID-19 disease, *n* (%)**	
Asymptomatic	8 (9.8)
Pauci-symptomatic	67 (81.7)
Hospitalised *	7 (8.5)

IQR: interquartile range; n: number; COVID-19: COronaVIrus Disease-2019; RA: rheumatoid arthritis; BMI: body mass index; DMARDs: disease-modifying antirheumatic drugs; TNF: Tumour Necrosis Factor; CTLA4-Ig: Cytotoxic T-Lymphocyte Antigen 4-Immunoglobulin; IL, interleukin; JAK: Janus kinase; -i: inhibitor; * 6 patients requiring low oxygen flux therapy.

**Table 2:** Baseline characteristics according to SARS-CoV-2 breakthrough infection after the third dose in 194 rheumatoid arthritis patients. Univariable and multivariable proportional hazard Cox regression analysis.

Characteristics	Uninfected	Breakthrough Infection	Cox Regression					
			Univariable			Multivariable		
			HR	95% CI	*p*	aHR	95% CI	*p*
**Overall**	112 (57.7)	82 (42.3)						
**Age in years, median (IQR)**	66 (57–72)	62 (53–71)	0.74 *	0.61–0.90	**0.003**			
**Age class under/over 50 years, *n* (%)**								
19–49	7 (31.8)	15 (68.2)	1			1		
50–87	105 (61.0)	67 (39.0)	0.41	0.24–0.73	**0.002**	0.38	0.20–0.74	**0.004**
**Gender, *n* (%)**								
Male	30 (52.6)	27 (47.4)	1					
Female	82 (59.9)	55 (40.1)	1.14	0.72–1.81	0.568			
**Presence of comorbidities, *n* (%)**								
No	30 (46.9)	34 (53.1)	1					
Yes	82 (63.1)	48 (36.9)	0.59	0.38–0.91	**0.018**	0.68	0.43–1.10	0.113
**BMI**	27 (23.5–28.6)	26 (22.7–28)	0.94	0.89–1.00	**0.050**	0.96	0.90–1.02	0.218
**Smoking habits, *n* (%)**								
No	94 (60.7)	61 (39.4)	1			1		
Yes	18 (46.2)	21 (53.8)	1.68	1.02–2.76	**0.040**	1.61	0.97–2.68	0.066
**Flu vaccination (season 2021/2022), *n*(%)**								
No	46 (56.1)	36 (43.9)	1					
Yes	66 (58.9)	46 (41.1)	0.92	0.59–1.42	0.703			
**Third-dose vaccine, *n* (%)**								
Comirnaty	110 (57.9)	80 (42.1)	1					
Spikevax/other	2 (50.0)	2 (50.0)	1.03	0.25–4.19	0.967			
**Rheumatoid arthritis disease**								
**Disease duration in months, median (IQR)**	126 (72–198)	120 (65–180)	1	1.00–1.00	0.956			
**Immunosuppressive drugs:** regimens containing								
**Conventional DMARDs, *n* (%)**								
No	68 (51.5)	64 (48.5)	1			1		
Yes	44 (71.0)	18 (29.0)	0.61	0.36–1.03	0.065	0.52	0.30–0.90	0.021
**TNFα-i, *n* (%)**								
No	88 (56.8)	67 (43.2)	1					
Yes	24 (61.5)	15 (38.5)	0.89	0.51–1.56	0.688			
**IL6-i, *n* (%)**								
No	105 (59.7)	71 (40.3)	1			1		
Yes	7 (38.9)	11 (61.1)	2.09	1.11–3.95	0.023	2.01	1.03–3.89	0.039
**CTLA4-Ig, *n* (%)**								
No	88 (56.4)	68 (43.6)	1					
Yes	24 (63.2)	14 (36.8)	0.68	0.38–1.22	0.200			
**JAK-i, *n* (%)**								
No	83 (57.2)	62 (42.8)	1					
Yes	29 (59.2)	20 (40.8)	0.89	0.54–1.48	0.657			
**CD20-i, *n* (%)**								
No	108 (59.0)	75 (41.0)	1			1		
Yes	4 (36.4)	7 (63.6)	1.74	0.80–3.78	0.162	2.88	1.27–6.51	0.011
**Corticosteroids, *n* (%)**								
No	90 (55.9)	71 (44.1)	1					
Yes	22 (66.7)	11 (33.3)	0.86	0.45–1.68	0.666			

IQR: interquartile range; BMI: body mass index; HR: hazard ratio; aHR: adjusted hazard ratio; CI: confidence interval; *n*: number; DMARDs: disease-modifying antirheumatic drugs; TNF: Tumour Necrosis Factor; CTLA4-Ig: Cytotoxic T-Lymphocyte Antigen 4-Immunoglobulin; IL, interleukin; JAK: Janus kinase; -i: inhibitor; * for 10 year increment.

**Table 3:** Cox regression analysis for the risk of SARS-CoV-2 infection in 41 RA patients according to B and T cell-specific immune responses.

				Univariable			Multivariable		
Characteristics	SARS-CoV-2 BI								
	No	Yes	*p*	HR	95% CI	*p*	aHR	95% CI	*p*
Total, ***n* (%)**	25 (61%)	16 (39%)							
**Immune responses**									
**anti-S/RBD (BAU/mL), *n* (%)**									
<809	7 (43.8)	9 (56.3)		1					
≥809	18 (72.0)	7 (28.0)		0.43	0.16–1.15	0.092	0.60	0.21–1.74	0.346
Neutralising antibodies, *n* (%) *									
<20	2 (25.0)	6 (75.0)		1			1		
≥20	22 (71.0)	9 (29.0)		0.27	0.10–0.74	**0.011**	0.36	0.12–1.07	0.067
**T cell-specific response detected by IFN-γ, *n* (%)**									
<16	10 (52.6)	9 (47.4)		1			1		
≥16	15 (68.2)	7 (31.8)	0.352	0.69	0.26–1.85	0.457	1.07	0.35–3.28	0.906

HR: hazard ratio; CI: confidence interval; *n*: number; anti-S/RBD: anti-spike receptor binding domain; BAU: binding antibody unit; IFN: interferon; BI: breakthrough infection; aHR: hazard ratio adjusted for time elapsed from third dose and sample collection. Regressions were fitted with robust standard error estimates. * Available in 39 patients.

**Table 4:** Characteristics associated with the severity of COVID-19 disease in RA patients with breakthrough infection.

	Hospitalisation			
	No	Yes	Total	*p*
**Overall**	**75 (91.5)**	**7 (8.5)**	**82 (100)**	
**Age, median (IQR)**	61 (53–71)	67 (52–80)	62 (53–71)	0.778
**Age class, over 50,** *n* (%)				1.000
19–49	14 (93.3)	1 (6.7)	15 (100)	
50–87	61 (91.0)	6 (8.9)	67 (100)	
**Age class, over 65,** *n* (%)				0.694
19–64	43 (93.5)	3 (6.5)	46 (100)	
65–87	32 (88.9)	4 (11.1)	36 (100)	
**Gender,** *n* (%)				
Female	51 (92.7)	4 (7.3)	55 (100)	0.679
Male	24 (88.9)	3 (11.1)	27 (100)	
**Comorbidities,** *n* (%)				0.127
None	33 (97.1)	1 (2.9)	34 (100)	
At least one	42 (87.5)	6 (12.5)	48 (100)	
**BMI, median (IQR)**	26 (23–28)	27 (20–27)	26 (22.7–28)	0.601
**Smoking habits,***n* (%)				0.274
No	57 (93.4)	4 (6.6)	61 (100)	
Yes	18 (85.7)	3 (14.3)	21 (100)	
**Flu vaccination (2021/2022),** *n* (%)				0.382
No	34 (94.4)	2 (5.6)	36 (100)	
Yes	41 (89.1)	5 (10.9)	46 (100)	
**Disease duration (months), median (IQR)**	120 (74–181)	60 (60–96)	120 (65–180)	**0.045**
**Regimens containing:**				
**Conventional DMARDs,** *n* (%)				0.645
No	59 (92.2)	5 (7.8)	64 (100)	
Yes	16 (88.9)	2 (11.1)	18 (100)	
**TNFα-i,** *n* (%)				1.000
No	61 (91.0)	6 (8.9)	67 (100)	
Yes	14 (93.3)	1 (6.7)	15 (100)	
**IL6R-i,** *n* (%)				0.586
No	64 (90.1)	7 (9.9)	71 (100)	
Yes	11 (100)	0 (0)	11 (100)	
**CTLA4-Ig,** *n* (%)				0.597
No	61 (89.7)	7 (10.3)	68 (100)	
Yes	14 (100)	0 (0)	14 (100)	
**JAK-i,** *n* (%)				1.000
No	56 (90.3)	6 (9.7)	62 (100)	
Yes	19 (95.0)	1 (5.0)	20 (100)	
**CD20-i,** *n* (%)				**0.001**
No	72 (96.0)	3 (4.0)	75 (100)	
Yes	3 (42.9)	4 (57.1)	7 (100)	
**Corticosteroids,** *n* (%)				1.000
No	65 (91.5)	6 (8.5)	71 (100)	
Yes	10 (90.9)	1 (9.1)	11 (100)	

IQR: interquartile range; BMI: body mass index; *n*: number; DMARDs: disease-modifying antirheumatic drugs; TNF: Tumour Necrosis Factor; CTLA4-Ig: Cytotoxic T-Lymphocyte Antigen 4-Immunoglobulin; IL, interleukin; JAK: Janus kinase; -i: inhibitor.

**Table 5:** Comparison of the characteristics studied between RA patients and HCWs.

Characteristics	RA	HCW	Total	*p*
Total, *n* (%)	194 (16.2)	1002 (83.8)	1.196 (100)	
**Age**	64 (54–71)	45 (34–54)	48 (36–57)	**<0.001**
**Gender,** *n* (%)				0.435
Female	137 (70.6)	679 (67.8)	816 (68.2)	
Male	57 (29.4)	323 (32.2)	380 (31.8)	
**Flu vaccination,** *n* (%)				**<0.001**
No	82 (42.3)	787 (78.5)	869 (72.7)	
Yes	112 (57.7)	215 (21.5)	327 (27.3)	
**Third dose vaccine,** *n* (%)				**<0.001**
Comirnaty	190 (97.9)	567 (56.6)	757 (63.3)	
Spikevax/other	4 (2.1)	435 (43.4)	439 (36.7)	
Breakthrough infection, *n* (%)				
Yes	82 (42.3)	530 (52.9)	612 (51.2)	**0.007**
No	112 (57.7)	472 (47.1)	584 (48.8)	

RA: rheumatoid arthritis; HCW: healthcare workers; *n*: number.

**Table 6:** Cox regression for the risk of SARS-CoV-2 infection in RA patients compared with HCW and the main characteristics.

Characteristics	Univariable			Multivariable		
	HR	95% CI	*p*	aHR	95% CI	*p*
**RA vs. HCW**	0.74	0.59–0.93	**0.012**	0.92	0.70–1.20	0.524
**Age (10 years increment)**	0.88	0.83–0.93	**0.000**	0.89	0.83–0.95	**0.001**
**Male vs. Female**	0.77	0.64–0.91	**0.003**	0.78	0.65–0.93	**0.007**
**Flu vaccination, yes vs. no**	0.96	0.80–1.15	0.636			
**Spikevax/others vs. Comirnaty**	1.09	0.93–1.28	0.314			

RA: rheumatoid arthritis; HCW: healthcare workers; HR: hazard ratio; aHR adjusted HR; CI: confidence interval.

## Data Availability

The raw data generated and/or analysed in the present study are available in our institutional repository (rawdata.inmi.it), subject to registration. The data can be found by selecting the article of interest from a list of articles ordered by the year of publication. No charge for granting access to data is required. In the event of a malfunction of the application, the request can be sent directly by e-mail to the library (biblioteca@inmi.it).

## Supplementary Materials

The following supporting information can be downloaded at: https://www.mdpi.com/article/10.3390/vaccines11111684/s1, Supplementary Figures S1–S3.
