# Supplementary material for: Older Age, a High Titre of Neutralising Antibodies and Therapy with Conventional DMARDs Are Associated with Protection from Breakthrough Infection in Rheumatoid Arthritis Patients after the Booster Dose of Anti-SARS-CoV-2 Vaccine
Source: Vaccines (Basel). 2023 Nov 2;11(11):1684. doi: 10.3390/vaccines11111684 (PMC10675674; doi:10.3390/vaccines11111684)
Supplement: Supplementary file 1 [file vaccines-11-01684-s001.zip › vaccines-2645479-supplementary.pdf]

Supplementary Figures

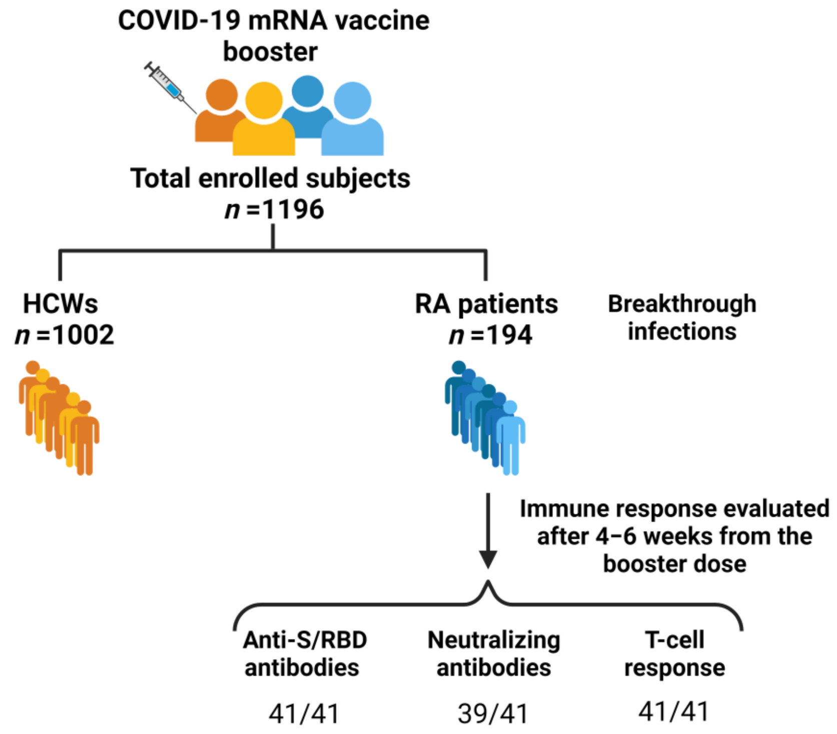

Figure S1. Flow chart of the enrolled subjects. A total of 1196 subjects were enrolled of whom 194 RA patients and 1002 HCWs. To evaluate the risk of breakthrough infection after the booster dose of COVID-19 mRNA vaccine, the enrolled HCWs and RA patients were followed up to 1 year. In a subgroup of the enrolled RA patients, the immune response was evaluated in terms of anti-S/RBD and neutralizing antibodies, and T-cell specific response after 4–6 weeks from the booster dose. Abbreviations: COVID-19, Coronavirus disease 2019; RA, rheumatoid arthritis; HCWs, health care workers; S, spike; RBD, receptor-binding domain. Created with BioRender.com.

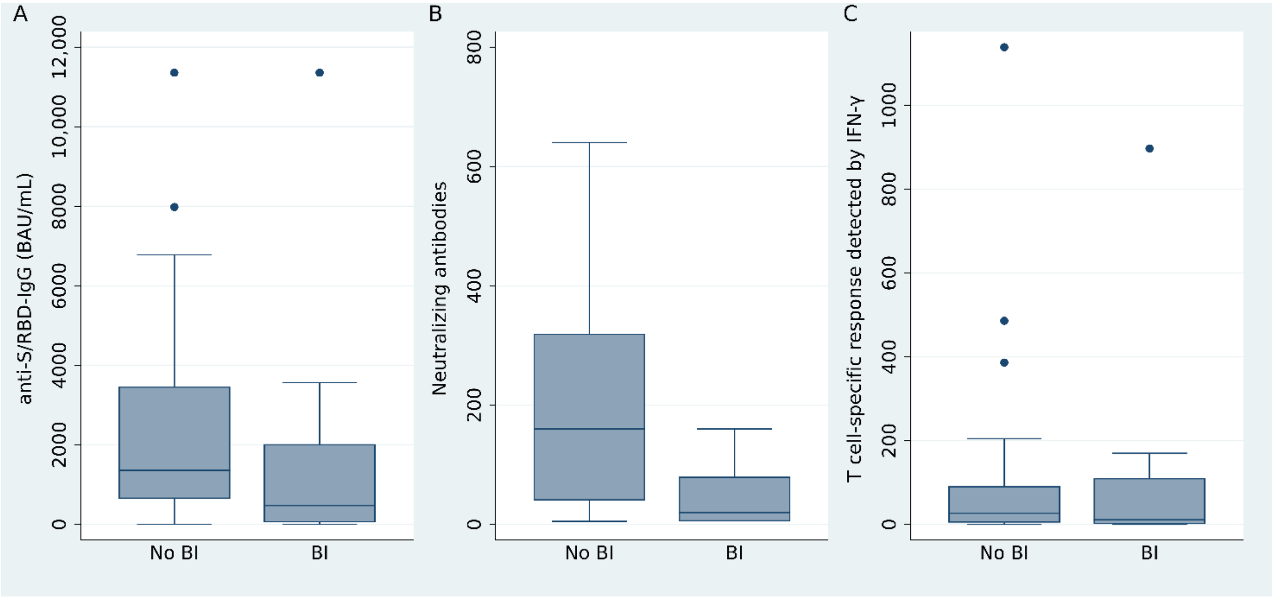

Figure S2. Distribution of the immune response levels according to infectious status in RA patients: breakthrough infection (BI) and non-breakthrough BI (no BI), for anti-S/RBD-IgG (BAU/mL) levels (A), neutralizing antibodies (B) and in T cell-specific response detected by IFN-γ (C). Abbreviations: anti-S/RBD: anti-spike receptor binding domain; BAU: binding antibody units; IFN: interferon.

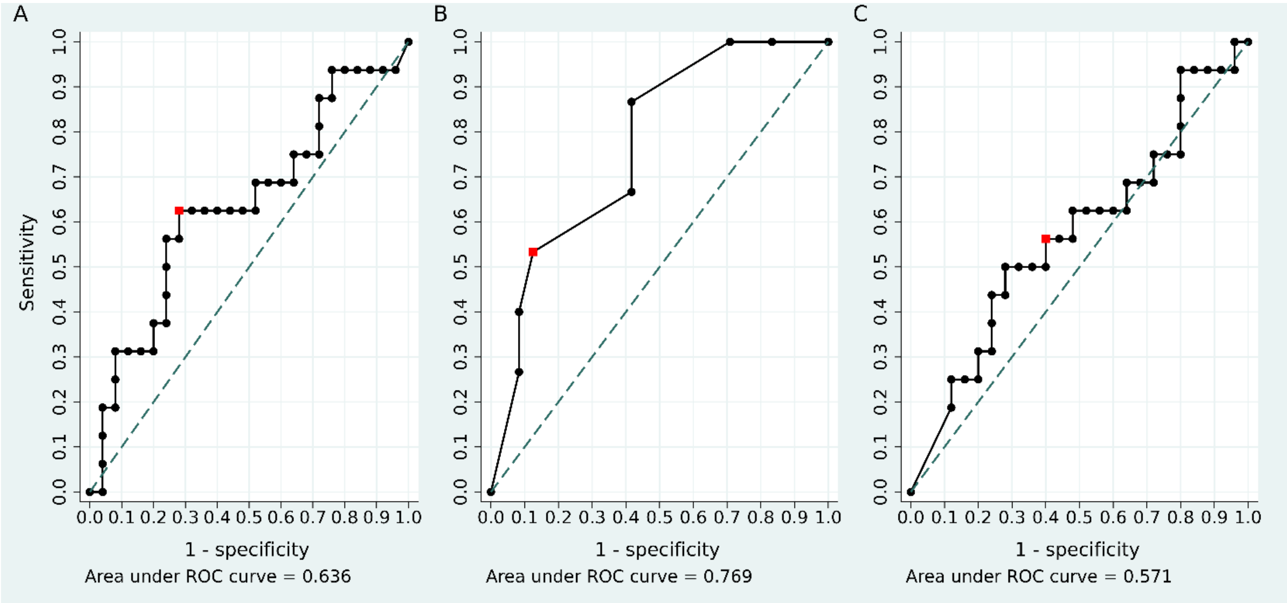

Figure S3. ROC curve analyses for evaluating immune response threshold for BI vs no BI after third dose vaccination. ROC curve for anti-S/RBD-IgG (BAU/mL) levels (A), neutralizing antibodies (B) and in T cell-specific response detected by IFN-γ (C). Abbreviations: ROC: Receiver operating curve; BAU: binding antibody units; IFN: interferon; anti-S/RBD: anti-spike receptor binding domain.
